# Supplementary material for: Allelic variation of the Tas1r3 taste receptor gene affects sweet taste responsiveness and metabolism of glucose in F1 mouse hybrids
Source: PLoS One. 2020 Jul 16;15(7):e0235913. doi: 10.1371/journal.pone.0235913 (PMC7365461; doi:10.1371/journal.pone.0235913)
Supplement: Attachment — Submitted filename: Response to reviewers.docx [file pone.0235913.s001.docx]

**PONE-D-20-05222**

**Revision 1**

**Responses to Reviewers #1 and #2**

**Response to Reviewer #1.**

**Reviewer #1:** In this manuscript, authors appeared the difference of sweet taste preference and sugar metabolism among the crossed mouse strains: F1 male hybrids obtained from 129SvPasCrl, B6 and/or B6-Tas1r3(-/-). This manuscript lacks the basic information about the sugar metabolism about 129Sv, B6, B6-Tas1r3(-/-). Moreover, there is a major problem with the description of discussion. Therefore, I recommend that this manuscript should be rewritten entirely.

**1.** Authors should appear the difference about the sugar metabolism between 129SvPasCrl and B6. The data of 129S, 129SB6F1, 129SB6-Tas1r3-/-F1, B6, B6-Tas1r3+/-, and B6-Tas1r3-/-should be shown in one graph.

**Response:**

Reviewer #1 insists on additional information but does not dispute the relevance of the main goal and provides no critique of experimental results, their evaluation, and our conclusions. We also consider the recommendation of Reviewer #1 “that this manuscript should be rewritten entirely” to be excessive.

The main goal of the current study was to define whether variations in a single taste receptor gene (*Tas1r3*) play a role in the regulation of glucose tolerance, insulin resistance, and some other parameters of metabolism. Developing the experimental model, we considered that the maintenance of glucose homeostasis involves a complex interplay of many genes and their actions in response to exogenous stimuli. Thus, the use of inbred mouse strains seems nonrelevant for definition of the role of the selected gene under physiological conditions. That is why the current study is based on comparisons between hybrids bearing different sets of *Tas1r3* dominant and recessive alleles but having identical background genotypes. We believe that the constant background genotype of hybrid F_1_ groups is an indispensable condition to rigorously characterize effects of *Tas1r3* polymorphisms on glucose homeostasis.

In this paradigm, “basic information about the sugar metabolism” (maybe glucose metabolism?) of the parental strains B6, B6-*Tas1r3-/-*, and 129 seems unnecessary to draw correct conclusions. Due to the polygenic nature of regulation of glucose homeostasis, parental strains would not be appropriate controls for polymorphic effects of the *Tas1r3* gene.

Furthermore, differences in glucose homeostasis (including glucose and insulin tolerance) between B6 and 129 inbred mouse strains and substrains are well characterized elsewhere (e.g., Almind and Kahn, 2004; Berglund et al., 2008; Toye et al., 2005; Goren et al., 2004; Kadota et al., 2016). One may also find the information in the Mouse Phenome Database of Jackson Laboratory (phenome.jax.org/projects/Eumorphia3/strains). In the literature, there are several examples of the effect of *Tas1r3* deletion on glucose metabolism regulation (e.g., Simon et al., 2014; Murovets et al., 2015).

At the same time, addressing comment 1 of Reviewer #1, we have added a supplementary graph (S1_Fig.) demonstrating glucose tolerance in C57BL/6J, C57BL/6ByJ, 129P3/J, 129Sv, and knockout B6-*Tas1r3-/-* mouse strains. The graph shows significant differences in responses between 129 and B6 inbred strains and overlap within substrains. Additionally, S1Fig. shows a significant impact of *Tas1r3* gene deletion.

Moreover, to address comment 1 and to make the paper clearer to nonspecialists, we have revised the Introduction section, by adding the following at line 119 of the Revised Manuscript with tracked changes: **“Studies have shown that plasma glucose level was considerably higher in the B6 strain than in 129 (Almind and Kahn, 2004; Berglund et al., 2008; phenome.jax.org/ projects/Eumorphia3/strains). Mice from the B6 strain show mild glucose intolerance and impaired insulin secretion despite normal insulin sensitivity (Toye et al., 2005). In contrast, the 129 substrains exhibited higher glucose tolerance (Almind and Kahn, 2004; phenome.jax.org/projects/Eumorphia 3strains). Likewise, the B6 mice were more insulin resistant during the insulin tolerance test, whereas 129SVE mice were relatively insulin sensitive (Almind and Kahn, 2004)”.**

To emphasize importance of the influences of the animals’ genetic background on the impact of genetic manipulation with a particular gene (Sittig et al., 2016), we also edited the conclusions by specifying the background genotype: at line 421: “In particular, presence of the dominant *Tas1r3* allele (*Sac^b^* locus) on the **129S2B6F1 background genotype** results in higher glucose tolerance, along with increased consumption and preference for sugars and artificial sweeteners during initial licking”.

**Reviewer #1 wrote:** “2. Discussion contains a lot of results. I think it is a place to discuss the cause of the difference among 129SB6F1, 129SB6-Tas1r3-/-F1, and B6-Tas1r3+/-.”

**Response:**

The guidelines for authors for *PLOS ONE* do not give specific recommendations on how to prepare the Discussion section. That is why we followed the Springer *Nature* author and reviewer tutorials (available at <https://www.springer.com/gp/authors-editors/authorandreviewertutorials/writing-a-journal-manuscript/discussion-and-conclusions/10285528)>. Following this guide, we did not duplicate raw data but discussed their genetic nature in connection with taste receptor gene *Tas1r3* allelic variations or effects of *Tas1r3* haplo-insufficiency. In the Discussion we also compared our results and conclusions with those from other investigations in this field and analyzed limitations of the study.

To address “the cause of the difference” between the assessed genotypes, we substantially expanded the Introduction section to include the following starting at line 50 of the marked-up manuscript:

“**There is good evidence that the sweet taste receptor proteins, along with several signal-transducing molecules, are expressed not only in taste buds on the tongue but also in organs regulating metabolism (Laffitte *et al*. 2014). Sweet taste receptor subunits T1R2 and T1R3 are coexpressed in enteroendocrine cells of the small intestine (Jang et al., 2007; Margolskee et al., 2007). Their activation by natural sugars and artificial sweeteners leads to secretion of gut hormones such as glucagon-like peptide (GLP-1), GLP-2, and the gastric inhibitory peptide, acting as incretins to enhance insulin secretion (Jang et al., 2007; Kokrashvili et al., 2009). In the intestine, T1R3 is also involved in regulation of expression of sodium-dependent glucose transporter isoform 1 (Margolskee et al., 2007). Coupling of T1R3 with T1R1 produces a heterodimer that serves as an intestinal L-amino acid sensor modulating cholecystokinin release (Daly et al., 2013).**

**The T1R3 protein is detected in mouse pancreatic β-cells, where its expression level is greater than T1R2, suggesting that most likely T1R3 is present in homodimeric form or is more sensitive to glucose heteromers when coupled with a calcium-sensing receptor (Kyriazis et al. 2012; Maitrepierre et al. 2012; Damak et al. 2003). In pancreatic** **β-cells, stimulation of the sweet taste receptor elicits insulin release by elevating intracellular Ca^2+^ and/or cAMP (Kojima and Nakagawa, 2011) synergetic to the major universal mechanism of metabolic detection of glucose, which includes the glucose transporter GLUT2, glucokinase, and the ATP-sensitive K^+^ (KATP) channel (Craig et al. 2008).** **In β-cells, T1R3 can be also dimerized with the T1R1 subunit, forming the receptor detecting amino acids, which stimulates insulin synthesis and reduces cell apoptosis, affecting the mechanistic target of rapamycin complex 1 (mTORC1) through activation of phospholipase C and extracellular signal-regulated protein kinase 1 (ERK1) and ERK2 (Wauson et al., 2012). Importantly, the knockout of T1R3 in mice resulted in substantial impairment of metabolism and cell proliferation. The *Tas1r3-/-* deletion decreased glucose tolerance (****Simon et al., 2014; Murovets et al. 2015) and increased insulin resistance (Murovets et al. 2014). T1R3 knockout animals are resistant to sucrose-induced obesity and have smaller fat depots when fed a high-sucrose diet (Simon et al., 2014). Deletion of *Tas1r3-/-* reduces activation of mTORC1 (Wauson et al., 2012) and decreases density of pancreatic islets and expression of caspase 3 (Murovets et al., 2019)”.**

Responding to **question 2**, Reviewer #1 evaluated the performed statistical analysis as inappropriate and non-rigorous.

We regret that the Reviewer gave no examples to support this statement. In performing the analysis of data from GTTs and ITTs we addressed the guidelines for mouse metabolic phenotyping in diabetes research, which specify that changes in glucose levels over time during a GTT or ITT are usually analyzed using two-way ANOVA, and AUCs are presented for GTT to draw reliable conclusions (Alquier and Poitout 2018). That is why we applied the ANOVA test in the current research, like we did in our similar paper published in *PLOS ONE* (Murovets et al., 2015).

**Question 4**

We are surprised at the negative answer of the Reviewer #1 to question 4, regarding quality of standard English. Of course, there are no native English speakers among the authors. That is why our papers are always checked by a native English speaker with appropriate background and experience, and the current manuscript is no exception. With Revision 1, we attach a supplementary file with a letter attesting to appropriate editing for the initial submission and the revision 1 (S2_Murovets_proofreading).

We believe that our answers to the comments will facilitate further impartial evaluation of the manuscript.

On behalf of the authors,

Vasiliy A. Zolotarev, Ph.D.

**Selected references**

Almind K, Kahn CR. Genetic determinants of energy expenditure and insulin resistance in diet-induced obesity in mice. Diabetes. 2004 Dec;53(12):3274-85.

Alquier T, Poitout V. Considerations and guidelines for mouse metabolic phenotyping in diabetes research. Diabetologia. 2018 Mar;61(3):526-538. doi: 10.1007/s00125-017-4495-9.

Berglund ED, Li CY, Poffenberger G, Ayala JE, Fueger PT, Willis SE, Jewell MM, Powers AC, Wasserman DH. Glucose metabolism in vivo in four commonly used inbred mouse strains. Diabetes. 2008 Jul;57(7):1790-9.

Goren HJ, Kulkarni RN, Kahn CR. Glucose homeostasis and tissue transcript content of insulin signaling intermediates in four inbred strains of mice: C57BL/6, C57BLKS/6, DBA/2, and 129X1. Endocrinology. 2004 Jul;145(7):3307-23.

Kadota Y, Kawakami T, Takasaki S, Sato M, Suzuki S. Gene expression related to lipid and glucose metabolism in white adipose tissue. Obes Res Clin Pract. 2016 Jan-Feb;10(1):85-93. doi: 10.1016/j.orcp.2015.04.009.

Sittig LJ, Carbonetto P, Engel KA, Krauss KS, Barrios-Camacho CM, Palmer AA. Genetic background limits generalizability of genotype-phenotype relationships. Neuron. 2016; 91:1253–1259.

Toye AA, Lippiat JD, Proks P, Shimomura K, Bentley L, Hugill A, Mijat V, Goldsworthy M, Moir L, Haynes A, Quarterman J, Freeman HC, Ashcroft FM, Cox RD. A genetic and physiological study of impaired glucose homeostasis control in C57BL/6J mice. Diabetologia 2005, 48(4), 675–686.

**Response to Reviewer #2.**

**Reviewer #2:** In the present report, the authors used the mouse F1 hybrids between 129S2 and B6, or between 129S and B6-Tas1r3-KO strains, and examined the effects of the allelic variation of the Tas1r3 gene on the sweet taste perception and metabolism of glucose. I have a few comments on the manuscript which should be addressed to strength their conclusions.

1. Introduction on p4: In 35th line, I think the B6 strain is “Sacb homozygote” not “Sacd homozygote”.

**Response:** Corrected, thank you.

2. The authors used the hybrids between B6By and 129 mice for Fig. 5a-c. The hybrid between 129S2 and B6 should be used here.

**Response:** The goal of the study was to elucidate whether polymorphisms of the single taste receptor gene affect carbohydrate metabolism.

The maintenance of glucose homeostasis involves a complex interplay of many genes and their actions in response to exogenous stimuli. To exclude polygenic effect on the phenotype and focus on effects of allelic variations of the single gene, we chose a hybrid F_1_ model with different sets of *Tas1r3* dominant and recessive alleles or that lacked the *Tas1r3* gene but had identical background genotypes.

Parental B6 substrains (C57BL/6J (B6) and C57BL/6ByJ (B6By)) carry the same *Tas1r3* dominant gene, while 129 substrains (129SvPasCrl (129S2) and 129P3/J (129P)) are *Tas1r3* recessive homozygotes. For this reason we consider it acceptable to use different parental substrains to illustrate the possibility of polymorphic effects. Please note that, in analyzing a particular trait, we used hybrids obtained from the same substrains, fully matching in background genotype.

On the other hand, an animal’s genetic background influences the impact of genetic manipulation with a particular gene. That is why, additionally, we compared glucose tolerance between parental substrains. Supplementary graph S1Fig. demonstrates that there are no differences in IP glucose tolerance between 129P and 129S2 substrains, or between B6 and B6By.

Line 297 of the Revised Manuscript with tracked changes now has the following addition:

“**Additional analysis of IP glucose tolerance was performed in parental strains: 129P, 129S2, B6, B6By, and B6-*Tas1r3-/-*. In the nonfasting state, parental strains differed in baseline glucose level, which was higher in the B6 strain, including the knockout substrain, than in 129 (S1Fig.). However, there was significant overlap between responses of B6 and B6By, as well as between 129P and 129S2. The IP glucose tolerance was substantially reduced in the B6 strain compared to 129, but no significant differences were found within strains excluding B6-*Tas1r3-/-*. The *Tas1r*3 gene deficiency markedly worsens glucose tolerance compared both to B6 and to B6By (S1Fig). Comparison of Fig. 3*a* and S1Fig. demonstrates that hybrid groups had intermediate glucose tolerance between parental strains.”**

3. T1R3 is also the important component of the umami taste receptor (T1R1+T1R3). The authors need to consider the effect on the umami taste receptor especially when they discuss the postoral events.

**Response:**

In the manuscript, we do not address mechanisms of metabolic control involving the T1R3 receptor protein. However, we fully agree with the Reviewer #2 that coupling of T1R3 with T1R1 in the small intestine or pancreas can specifically activate intracellular signaling cascades regulating hormone secretion and cell proliferation. To emphasize it, we included the following references in the revised manuscript.

Line 58 of the marked-up manuscript: “**Coupling of T1R3 with T1R1 produces heterodimer that serves as an intestinal L-amino acid sensor modulating cholecystokinin release (Daly et al., 2013)”.**

Line 67 of the marked-up manuscript: “**In β-cells, T1R3 can be also dimerized with the T1R1 subunit, forming the receptor detecting amino acids, which stimulates insulin synthesis and reduces cell apoptosis affecting the mechanistic target of rapamycin complex 1 (mTORC1) through activation of phospholipase C and extracellular signal-regulated protein kinase 1 (ERK1) and ERK2 (Wauson et al., 2012). ... Deletion of Tas1r3-/- reduces activation of mTORC1 (Wauson et al., 2012) and decreases density of pancreatic islets and expression of caspase 3 (Murovets et al., 2019)”.**

Sincerely,

On behalf of the authors,

Vasiliy A. Zolotarev, Ph.D.
