# Supplementary material for: Allelic variation of the Tas1r3 taste receptor gene affects sweet taste responsiveness and metabolism of glucose in F1 mouse hybrids
Source: PLoS One. 2020 Jul 16;15(7):e0235913. doi: 10.1371/journal.pone.0235913 (PMC7365461; doi:10.1371/journal.pone.0235913)
Supplement: Attachment — Submitted filename: Response to Reviewer.docx [file pone.0235913.s002.docx]

**PONE-D-20-05222R1**

**Revision 2**

**Responses to Reviewers**

**Response to Reviewer #1.**

**Reviewer #1 wrote:** “I was able to understand that the glucose tolerance of 129 and B6 is completely different since authors provided Figure S1. I would like Figure S1 to be included in the main text for general readers of PLOS ONE”.

**Response:** We agree with the reviewer that addition of figure demonstrating glucose tolerance in parent strains could be useful for general readers. For this purpose, we inserted Fig. 3 and edited Table 2 (L. 295 of the Revised manuscript with tracked changes): **“Initial analysis of IP glucose tolerance was performed in parental strains: 129P, 129S2, B6, B6By, and B6-*Tas1r3-/-*. In the nonfasting state, parental strains differed in baseline glucose level, which was higher in the B6 strain, including the knockout substrain, than in 129 (Fig. 3; p<0,05). However, there was significant overlap between responses of B6 and B6By, as well as between 129P and 129S2. The IP glucose tolerance was substantially reduced in the B6 strain compared to 129 (p<0.005), but no significant differences were found within strains excluding B6-*Tas1r3-/-*. The *Tas1r3* gene deficiency markedly worsens glucose tolerance (Fig. 3)** **compared both to B6 and to B6By (p<0.01).**

**Comparison of Fig. 3 and Fig. 4a demonstrates that hybrid groups had intermediate glucose tolerance between parental strains.”**

**The Reviewer #1:”** Is it correct to assume that the glucose metabolism of 129B6 and B6 is also different? I recommend that authors discuss about the validity of comparison between the data from 129B6 and 129B6-T1r3KO and the data form B6 and B6/B6-T1r3KO, even though the physiological background of 129B6 and B6/B6 are not equivalent”.

**Response:** Yes, it is correct. For this reason, we did not compare 129B6-*Tas1r3*KO with B6-*T1r3+/-*. Distinctions between 129B6 and 129B6-*Tas1r3*KO hybrids were analyzed to assess effects of dominant and recessive alleles of the *Tas1r3* gene. B6 and B6-*Tas1r3+/-* mice differed in number of dominant *Tas1r3* genes. Using B6 and B6-*Tas1r3+/-* groups, we primarily evaluated the effect of the *Tas1r3* gene dosage (hemizygosity). In the available literature, there are no data on interaction between *Tas1r3* and other genes that supports validity of comparison between groups with identical B6 genetic background and, to some extent, allows to transfer results to 129B6 hybrids.

**Reviewer #1:** “As authors mentioned, the results are not written twice. However, I think that some sentences should be written in results (L.337-352, L404-410)”.

**Response:** In the manuscript, we tried to keep the following scheme: the Results section describes responses of hybrids, whereas in the Discussion section we analyze effects of the *Tas1r3* genotype and compare the obtained data with literature. That is why we would like to leave mentioned phrases in place.

**Reviewer #1:** In addition, more discussion of the function of the polymorphisms in T1r3 should be needed. As for the additional information in introduction (L.50-L.76), I think it is necessary to discuss how these molecules are affected by polymorphisms in T1r3.

**Response:** To our knowledge, almost all publications discussing effects of the mouse gene *Tas1r3* polymorphisms address to the detailed analysis performed by Danielle Reed et al. (2004), which we cited in the current manuscript. We edited new revision by adding the following (L. 95): “Allelic variants of the *Tas1r3* gene correspond mainly to three nonsynonymous single nucleotide polymorphisms (SNPs) **which do not act by blocking gene expression, changing alternative splicing, or interfering with protein translation in taste tissue. Among the polymorphisms, T179C, which causes a substitution of isoleucine to threonine at position 60 in the extracellular N domain of the T1R3 protein, influence the ability of the protein to form dimers or bind sweeteners (Reed et al. 2004)”.** Further we wrote (L. 100): “This substitution reduces *in* *vitro* binding of T1R3 to caloric or noncaloric sweeteners, increasing, for instance, effective dose for sucrose up to 1000% (Nie *et al.* 2005).”

**Reviewer #1:** I would like authors to explain the sentences of L.383 to L.387 to correspond the introduction (L.50-L.76).

**Response:** When glucose is applied intragastrically in the GTT, sweet taste receptors in the small intestine are involved in reinforcement of secretion of gut hormones GLP-1 and GIP, which in turn facilitate insulin output in the pancreas and up-regulate expression of the intestinal glucose transporter SGLT1. To clarify it, we edited the Introduction section (L. 57): “In the intestine, T1R3 **upregulates** **through GLP-1 and GIP** expression of sodium-dependent glucose transporter isoform 1 **(SGLT1) important for the provision of glucose to the body and avoidance of intestinal malabsorption** (Margolskee et al., 2007)”. When glucose is applied intraperitoneally, it affects sweet taste receptors in the pancreas stimulating insulin production but does not interact with intestinal receptors. We believe that impairment of T1R3-mediated sensitivity in B6-*Tasr3+/-* can both elevate plasma glucose level due to reduction of insulin output and suppress glucose absorptive capacity of the intestine. Thus, in hemizygotes after intraperitoneal load with glucose, reduced intestinal absorption can be discussed as a reason of lower plasma glucose level found in our experiments (L. 386): “B6-*Tas1r3+/-* hemizygotes exhibited lower plasma glucose concentrations, which can reflect reduced expression of **SGLT1 because of** **decreased GLP1** **and GIP** production in the small intestine mucosa (Margolskee *et al.* 2007; Sigoillot *et al.* 2012), rather than a consequence of impaired utilization of glucose in tissues”. However, additional studies of this phenomenon are needed beyond the scope of this article.

We believe that translational value of our findings is that they show for the first time that allele variant of the *Tas1r3* gene alter *in vivo* glucose metabolism in mice, and that similar relationships may exist in humans.

Sincerely,

On behalf of the authors,

Vasiliy Zolotarev, Ph.D.
